# Postural displacement induced by electrical stimulation; A new approach to examine postural recovery

**DOI:** 10.1371/journal.pone.0273282

**Published:** 2022-08-18

**Authors:** Behdad Tahayori, Bahman Tahayori, Alireza Mehdizadeh, David M. Koceja

**Affiliations:** 1 Department of Physical Therapy, University of Saint Augustine, Miami FL, United States of America; 2 The Florey Institute of Neuroscience and Mental Health, The University of Melbourne, Heidelberg, VIC, Australia; 3 Department of Medical Physics, Shiraz University of Medical Sciences, Shiraz, Fars, Iran; 4 Department of Kinesiology and Program in Neural Sciences, Indiana University, Bloomington, IN, United States of America; Toronto Rehabilitation Institute - UHN, CANADA

## Abstract

**Background:**

Controlling upright posture entails acute adjustments by the neuromuscular system to keep the center of mass (COM) within the limits of a relatively small base of support. Sudden displacement of the COM triggers several strategies and balance recovery mechanisms to prevent excessive COM displacement.

**New method:**

We have examined and quantified a new approach to induce an internal neuromuscular perturbation in standing posture on 15 healthy individuals to provide an insight into the mechanism of loss of balance (LOB). The method comprises eliciting an H-reflex protocol while subjects are standing which produces a contraction in soleus and gastrocnemius muscles. We have also defined analytical techniques to provide biomarkers of balance control during perturbation. We used M-Max unilaterally or bilaterally and induced a forward or sideway perturbation. The vector analysis and the Equilibrium Point calculations defined here can quantify the amplitude, direction, and evolution of the perturbation.

**Results:**

Clear patterns of loss of balance due to stimulation was observed. Compared to quiet standing, the density of the EPs substantially increased in the perturbation phase. Leftward stimulation produced significantly higher number of EPs compared to the bilateral stimulation condition which could be due to the fact that the left leg was the nondominant side in all our subjects.

**Comparison and conclusion:**

In this study we provide a proof-of-concept technique for examining recovery from perturbation. The advantage of this technique is that it provides a safe perturbation, is internally induced at the spinal cord level, and is free from other factors that might complicate the recovery analysis (e.g., locomotion and the integration of the spinal pattern generator and cutaneous pathways in mediating changes). We have shown that the perturbation induced by this method can be quantified as vectors. We have also shown that the density of instantaneous equilibrium points (EPs) could be a good biomarker for defining and examining the perturbation phase. Thus, this protocol and analysis provides a unique individual assessment of recovery which can be used to assess interventions. Finally, given that the maximal motor response is used as the perturbation (e.g., M-max) it is highly reliable and reproducible within an individual patient.

## Introduction

Loss of balance and subsequent over ground fall is a major cause of disability and death among elderly people in the United States [1,2]. Elderly people’s falls are notoriously dangerous; up to 60% of falls in elderly population result in severe physical injuries. Recent years statistics show that more than 50% of fall patients are being placed in long-term care and nursing facilities [3]. However, replicating an actual fall in a laboratory environment is challenging because it is and involuntary and—most of the time—unpredictable event. Nonetheless, understanding the neural and mechanical events occurring during loss of balance (LOB) are helpful to understand the underlying causes/mechanisms of fall. The mechanism of perturbation determines the type of responses and events leading to LOB and fall [4]. Therefore, the findings might not be generalizable to all falls, rather, might be comparable with falls with similar perturbation mechanisms. For this very reason, having different experimental methods to examine loss of balance would increase our understanding on different aspects of LOB and possibly falls.

There are three broad categories of inducing an experimental loss of balance: (a) applying an external force/torque to the torso [5], (b) using moving surfaces to perturb standing balance [6] and (c) self-generating perturbation such as moving body parts [7]. The problem of “awareness” about the imminent loss of balance is highly pronounced in self-generating perturbations and triggers feedforward and open loop anticipatory mechanisms for preventing the fall [8,9]. This method is, therefore, mainly applicable for understanding preventative and anticipatory mechanisms in response to loss of balance but might not be sensitive for spontaneous over-ground falling. Moving surface protocols closely replicate loss of balance and falling on slippery surfaces. There are a cascade of response mechanisms to this type of perturbation [10] but the results might not be generalizable to spontaneous loss of balance due to aging [11].

Impacting the torso causes a sudden displacement of the COM over the base of support (BOS). LOB can be accentuated by the opposite direction of movement of COP and COM. A common feature among all these methods is that all the protocols intend to bring–unexpectedly–the COP to the limits of BOS. The difference is mainly in the level of response activation.

Therefore, different methods provide insights on distinct aspects of loss of balance and potentially, falling. However, there are other ways for inducing perturbation or examining the role of sensory input for control of posture. As an example, Duclos et al. induced proprioceptive perturbation through vibration of the Achilles tendon [12].

As an add-on to the existing methods, here we introduced and tested a new approach and analytical method for examining loss of balance. The core idea is to elicit electrically evoked responses in the muscles through H-reflex method/pathway in upright standing, causing contraction in postural muscles and examining the behavior of COP movement along with the neuromuscular responses. Using the M-max is technically less challenging as it is easier to have consistency in its amplitude among different trials.

The loss of balance and falls is inherently a multi-faceted event; this method can potentially provide new information and biomarkers for certain loss of balance recovery mechanisms. Since this method triggers the LOB through a sudden muscle twitch, it can better define neuromuscular response to perturbation, as the cause of perturbation lies within the neuromuscular system and does not initiate the LOB by displacing the COM. In cases of moving surfaces, as an example, there is a displacement of COP, followed by reactive-corrective joint movement, whereas in our method, there is a perturbing joint moment, followed by reactive-corrective joint moment. Both of these phases of perturbing moment and reactive-corrective moment can be defined through the vectors of COP movement. The initial phase of COP displacement (what we regards as forward vectors) corresponds to the perturbation, and the second phase of backwards movement corresponds to corrective movements caused by ankle moments. As such, this method defines and measures both the perturbation and recovery phases of LOB caused by the same neuromuscular system.

In normal, unperturbed upright standing, COP migration is a reflection of the postural control system to maintain its equilibrium within the base of support [13]. A theory developed by Zatsiorsky and Duarte in 1999 suggesting that the COP oscillates around an attracting point (referred to as equilibrium points—EPS), whereby it constantly makes corrections to maintain the equilibrium [14]. Being inspired by this theory, we measured the density (number of EPS per second) of these equilibrium points in quiet standing and in the perturbation phase. Since the postural control system is being moved out of its so-called stable position, we anticipated the during the perturbation phase, the density of EPS would increase suggesting that the postural control system is exploring to find its stable status and thereby rapidly moving from one EP to another. Since in our method the initiation of perturbation is consistently detectable, determining the density of the EPS in the perturbation phase is easily attainable.

Our lab has a long history of examining the H-reflex pathway during upright standing [15,16], with a special interest on investigating postural control in elderly with risk of falling. The past studies published from this lab were mainly focusing on the neural correlates of fall risk, whereas here, we developed a newer look and analysis on the mechanical effects of nerve stimulation during standing.

## Method

### New approach

The method entails eliciting the H-reflex protocol in upright standing, causing a single twitch in soleus-gastrocnemius muscles. Soleus H-reflex protocol has been explained in detail in (Knikou 2008) and its functional relevance has been well-documented [15–18]. Briefly, a short duration pulse is applied on the posterior tibial nerve, stimulating sensory and motor fibers resulting in a direct motor response (upon stimulation of motor fibers) and a spinal response (upon stimulation of sensory fibers, mainly Ia fibers). By increasing the stimulation intensity, the H-reflex amplitude decreases due to collision of the impulses inside the motor fiber and eventually a maximum muscle response (M-Max) appears with no H-reflex [19–21]

An M-max in Gastrosoleus muscle in standing causes a sudden displacement in the ankle joint and hence a perturbation in standing posture. This type of perturbation is caused by a sudden postural muscle twitch and therefore, can be regarded as an internal perturbation. Unlike applying a mass or a push near to the center of mass of subjects, this perturbation is close to the hinge of the inverted pendulum model, simulating an ankle strategy for balance corrections. COP and force data are good indicators for quantifying the perturbation.

The stimulation, and therefore, perturbation, can be applied unilaterally on one leg or bilaterally on both legs at the same time.

This method, therefore, needs a nerve stimulation device, a forceplate, a surface EMG system and a data collection hardware and software.

### Analysis approach

By applying this method, a clear deflection in COP movement appears after each stimulation. The initial deflection peak is usually followed by a series of smaller amplitude deflections in COP movement. These oscillatory movements tend to bring the system back to its normal equilibrium [22]. This pattern of a large initial deflection followed by a train of small corrections is a manifestation of a closed-loop control of posture [23]. As such, the analysis of a normal response provides features for comparison with pathological conditions.

**Fig 1:**
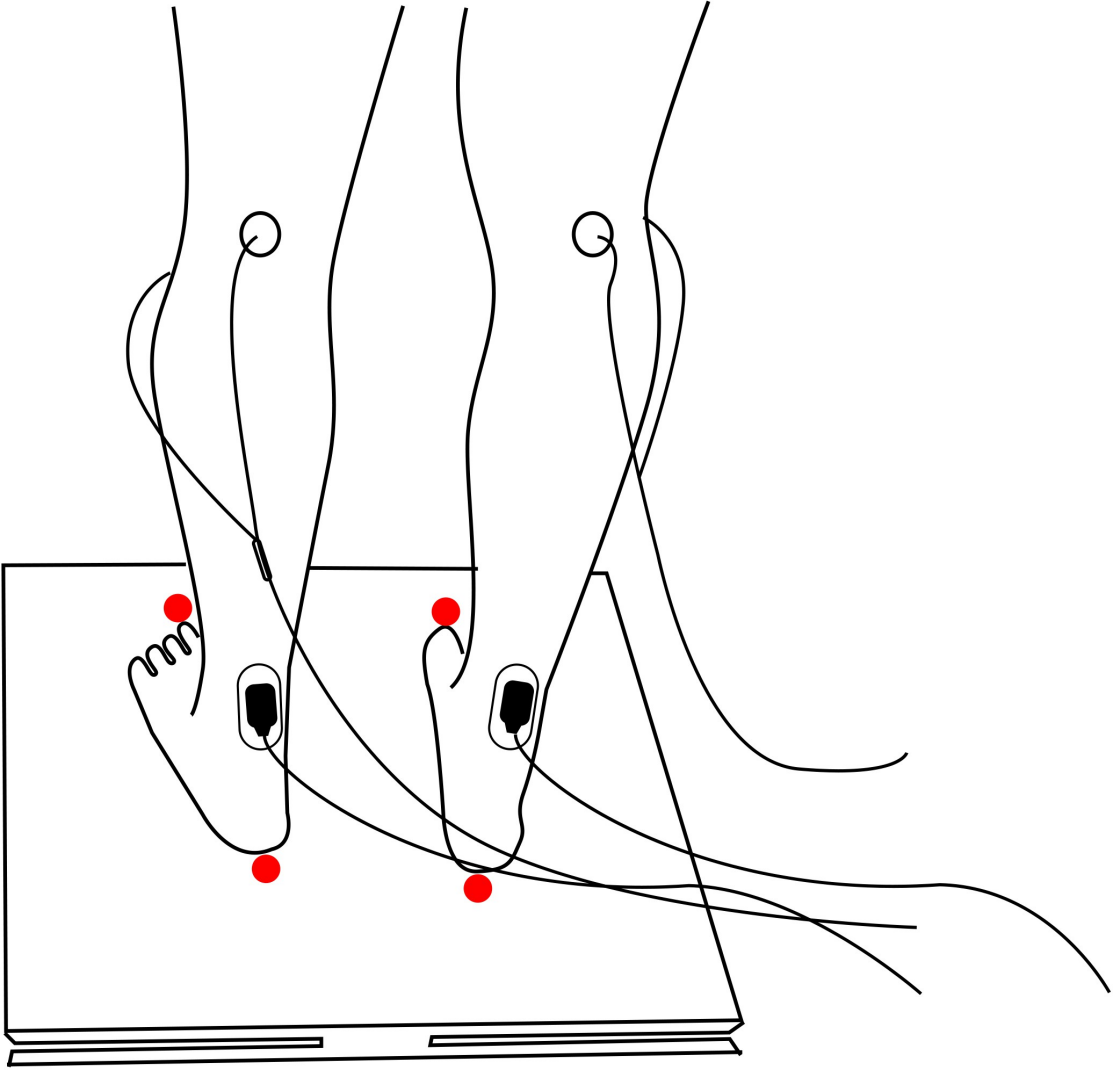
Protocol setup. Stimulation electrodes were placed on the posterior tibial nerve to elicit an M-wave and or H-reflex. The response was recorded by surface electrodes placed on the soleus muscle. The resultant muscle contraction would induce a plantar flexion moment in a closed kinematic chain, causing a perturbation in standing posture. Unilateral or bilateral stimulation would provide sideway or forward perturbations. Red dots represent the places marked for measuring BOS and feet locations.

A sampler COP deflection pattern in response to stimulation is shown in Fig 1.

To quantify the duration of perturbation and determine the instant at which COP movement became stable again, we used a method inspired by the Rambling-Trembling hypothesis [14,24]. Our assumption is that these deflections are occurring to bring the COP back to its normal equilibrium points and for that reason, several corrections from the CNS are being applied by moving the COP back and forth (small amplitude overcorrections following a large size perturbation). Therefore, there expects to be a change in the number of equilibrium points in the perturbation zone. It is suggested that during postural instability, the control mechanism of posture increases its search for stable points [25]. We, therefore, expect to observe more instantaneous equilibrium points, as the CNS is trying to get back to stability. The equilibrium points were determined from the following formula:

$$\left\{ \begin{array}{l} EP_{X}(i)=COP_{x}(i)|F_{x}(i)\;x\;F_{x}(i‐1)<0\\ \;\mathrm{and}\\ EP_{Y}(i)=COP_{Y}(i)|F_{Y}(i)\;x\;F_{Y}(i‐1)<0 \end{array}\right.$$

Where EP is the Equilibrium Points, COP is the center of pressure and F_x_ and F_y_ is horizontal force from the forceplate. EPs for Anterior-Posterior (AP) and mediolateral (ML) directions were calculated separately.

The density of EPs was measured in the unperturbed trial and an average of EP per second was calculated. EP density with bins of 1 sec duration with a 500 ms overlap window was calculated after each perturbation. The termination of the perturbation based on COP movement is a challenging task as the COP is a nonstationary signal. To estimate the termination of perturbation, we considered the density of EP getting back to the pre-stimulation state. However, this would result in time spans which were skeptical by visual inspection. We estimated the termination of perturbation through visual inspection on 3 subjects and took an average of the resultant EP density at the cutoff instance. This value on average was about 85%. We, therefore, used 85% as the cutoff.

Each perturbation can be quantified as a vector with the length of the vector being the size of COP displacement and the angle of the vector being the angle of deviation of the COP. To calculate these vectors, the initial point of the vector was regarded as the average location of COP for 2 seconds prior to the instant of stimulation delivery and the endpoint was regarded as the peak deflection (turning point) of the COP.

The angle of vectors is affected by the orientation and foot placement of the subject on the forceplate. To correct for this confounding factor, feet locations were marked on the forceplate and after the subject stepped off the plate, the placement of these marks were pressed by a metal rod, one at a time, and recorded on a separate file. These points were used to define feet angle. Angle bisectors of the feet angles were calculated and the perturbation vectors were rotated relative to the angle bisector.

The vectors can be resolved to two orthogonal components, corresponding to ML and AP trajectory of COP during perturbation. We have observed that this correction did not make a significant change in the direction of force vector when we compared them with uncorrected vectors. That is because our subjects stood relatively in the same configuration in different trials. However, if subjects stand on the forceplate at different angles, this would substantially change the vectors relative to the coordinate systems of the forceplate.

### Subjects and testing protocol

Fifteen healthy young individuals (age: 26.1±3.2, 9 males, 6 females, height: 1.67±0.34, Weight: 64.2±4.1) who reported to be free of any orthopedic, visual, or neurological disorders participated in this study. This study was approved by the Institutional Review Board (IRB) of Indiana University Bloomington (IRB number: 1204008477-A005). All subjects signed the written informed consent form prior to participating in the study. Subjects stood barefoot on an AccuSway forceplate (AMTI Inc, Watertown, MA, USA) without any instruction to change their usual posture and looked at a target 3 meters in front of them, at their eye level. An initial 60 second unperturbed trial was recorded to measure normal movement of COP with no perturbation.

The M-max was elicited while subjects were standing comfortably on the forceplate. Total of fifteen stimulations were delivered with minimum intervals of 16 seconds (20 sec ± 4 sec) to ensure that the subjects had fully recovered from perturbation. Stimulations were randomly given either to the right, left or both legs simultaneously. As such, a total of 5 stimuli were delivered for each side (right, left, both). We made the rest interval variable (from 16 s to 24 s) so that the subjects would not predict the time of stimulation.

The electromyography (EMG) activity of the Soleus and Tibialis Anterior (TA) muscles of both legs were recorded using surface EMG electrodes (Therapeutic Unlimited, Company dissolved). Electrodes were placed on the bulk of the muscles, parallel to muscle fibers. The Soleus EMG signal was used to observe and define the M-wave responses. We monitor TA EMG activity to ensure that subjects were not co-contracting their muscles and hence changing their response strategies to perturbation.

Stimulations were delivered through a DS7A constant current stimulator (Digitimer, Welwyn Garden City, Hertfordshire, UK) with a pulse width of 1 ms. A stainless-steel cathode disc electrode was placed at the back of the knee on the posterior tibial nerve. The anode electrode was placed proximal to the superior pole of the patella. The amplitude of M-wave and the h-reflex is position dependent. We, therefore, determined M-max while the subject was standing, but prior to initiating the experimental trials. The intensity of the pulses was increased until a saturated M-response (M-max) was observed. Care was given as to avoid contamination of the fibular nerve [26]. This was verified by a strong contraction of the soleus and no contraction in the peroneal muscles.

Forceplate, EMG data and stimulation timestamps were fed into an Analog to Digital Converter (NI-6211-USB, National Instrument, Austin, Texas, USA) and digitized at 4000 samples per second. A customized program written in DASYLab (Measurement Computing Corporation, Norton, MA, USA) was used to observe the signals, measure M-max values in real-time and save data on a computer for further offline analysis.

Fig 1 is an illustration of the setup, showing the standing position with stimulation and recording electrodes in place.

The COP signal was calculated using the conventional method provided by the manufacturer. The instants of perturbation were extracted from the timestamps of the stimulator machine.

A part of the offline analysis was performed in custom-written programs with MATLAB R2020a (MathWorks, Natick, MA, USA). Certain analyses were performed in Python.

To compare the amplitude of M-max and EP density at three different stimulation conditions, we conducted an analysis of variance (ANOVA) to examine the overall differences among groups and performed Bonferroni post-hoc tests for pairwise comparison.

## Results

**Fig 2:**
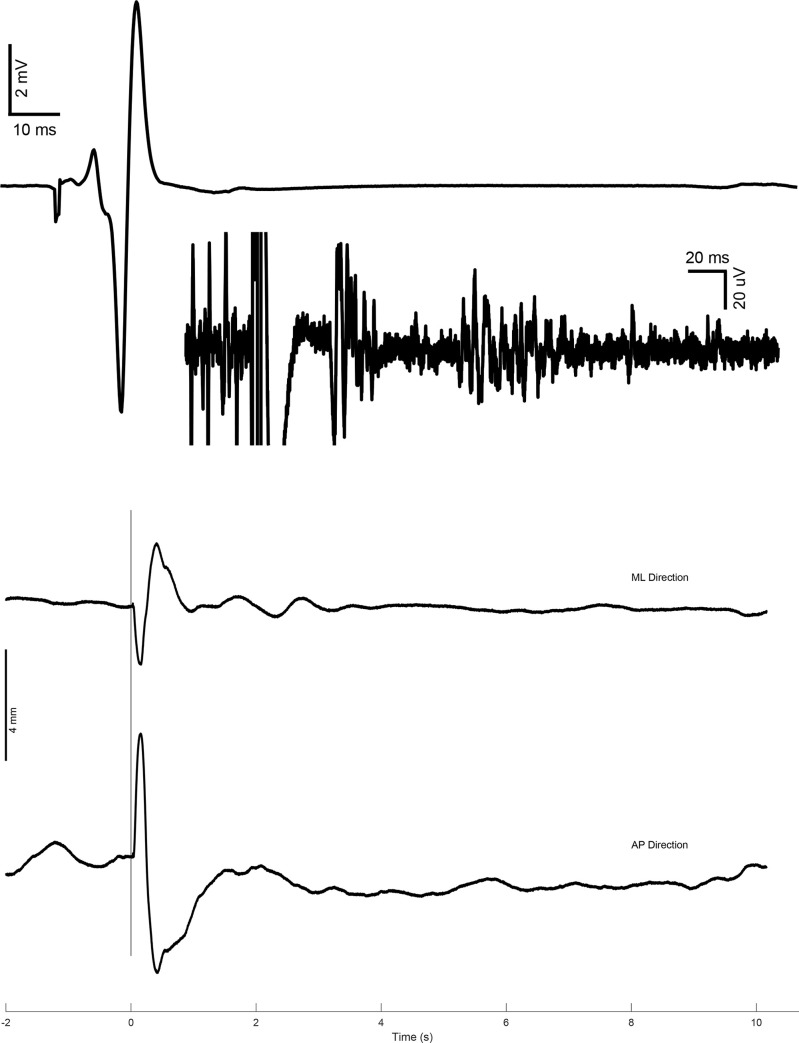
(A) Sample M-max with almost no observable H-reflex. The inset is an amplification of the same signal to show EMG activity, (B) Sample of COP trajectory deflection.

The electrical stimulation of the posterior tibial nerve elicited an M-max in the corresponding Soleus muscle and induced a perturbation in subjects’ posture. This was manifested by a sudden deflection of COP trajectory. A sampler EMG response including M-max and subsequent EMG activity is shown in ‘Fig 2A. To better observe the EMG signal after the artifact caused by stimulation, the signal was scaled to uV and is shown in the inset of panel A. COP deflection as a result of this M-wave is presented in Fig 2B.

**Fig 3:**
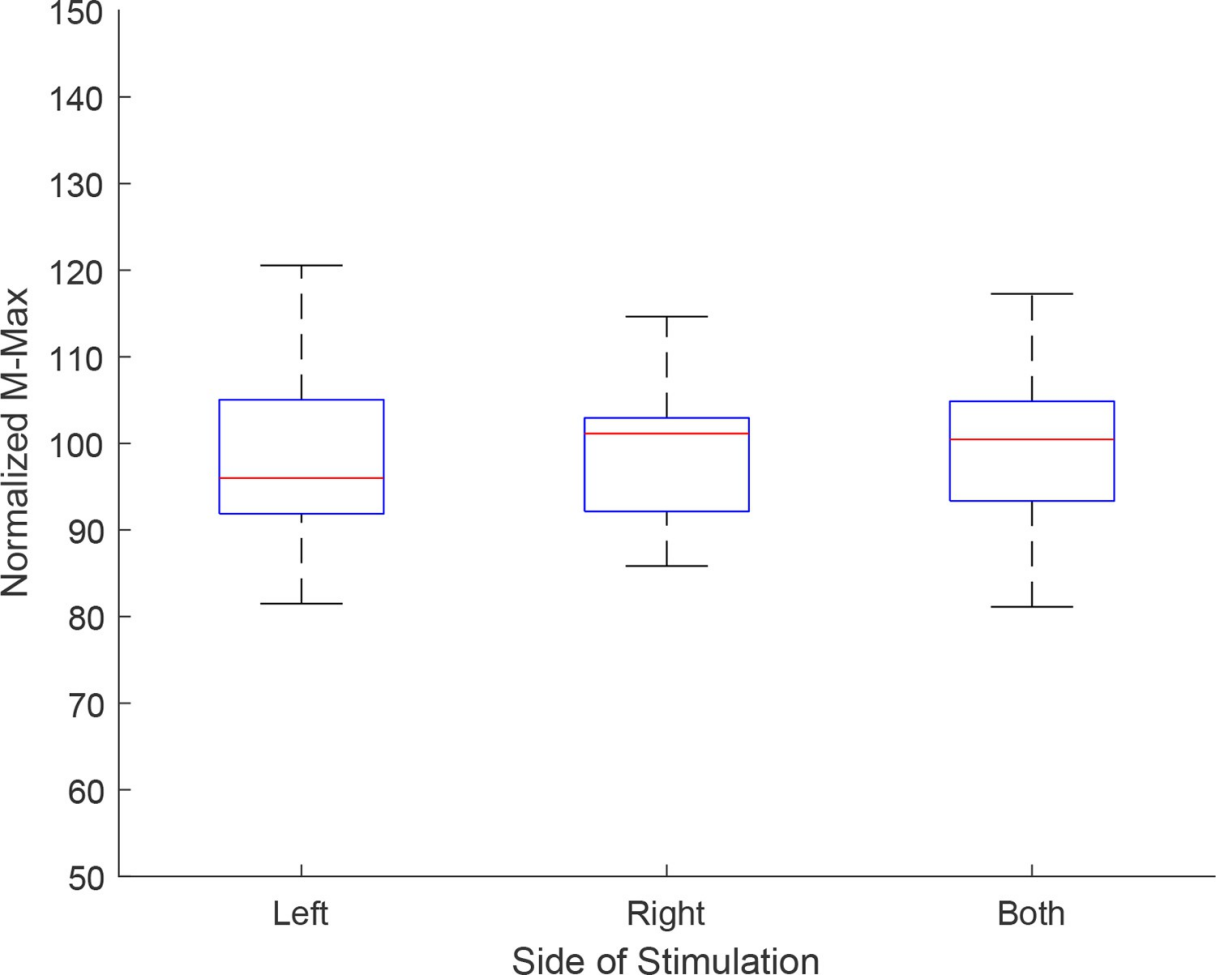
M-max amplitude was monitored for consistency of stimulation. Normalized values of M-max (each value divided by its mean), is shown here for each side of stimulation. The box plot shows the mean and standard deviation for all subjects in each condition. No statistical differences were observed among these three conditions.

The amplitude of M-max was monitored after each stimulation to prevent any significant change in the response and inconsistency in the perturbation caused by this stimulation. Fig 3 shows the peak-to-peak amplitude of M-waves in a sampler subject, showing a normal fluctuation in the amplitude but no substantial drift. The coefficient of variation for M-max was on average 11.34, 9.35 and 10.53 for left side, right side and both side stimulation conditions.

### Equilibrium points

**Fig 4:**
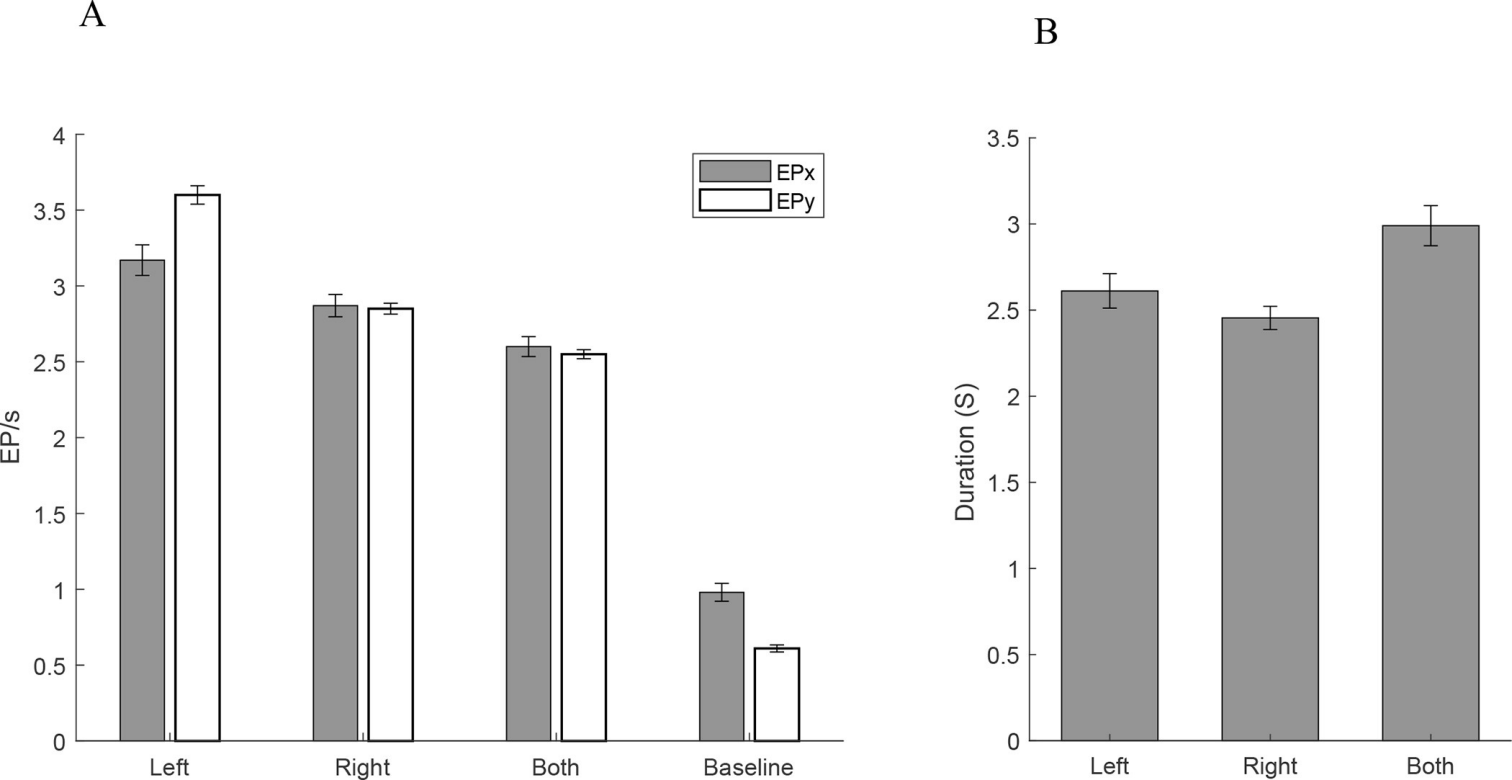
(A) Density of Equilibrium points in the AP and ML directions. Leftward perturbation (stimulation of the left side) caused a higher density of EPs in the perturbation phase. This was statistically significantly higher than the bilateral stimulation condition. Statistically significant differences were found between left and right side stimulation for both EPx and EPy and between left and bilateral stimulation. (B) Duration of perturbation. The error bars are Standard Error of the Means.

The density of Equilibrium Points (EPs) was calculated in the quiet standing and in the perturbation period. The analysis of Variance showed a significant overall difference among the four conditions (F_3,56_ = 47.46, P<0.001). Post-hoc Bonferroni analysis showed that the number of EPS/s significantly increase with the stimulation (regardless of the side). It also showed that the left side stimulation significantly increases the number of EPSx and EPSy per second compared to the right side (p = 0.006, p = 0.081 respectively with α = 0.0125). Similar differences were observed comparing left and bilateral stimulation(p = 0.004, p = 0.061 respectively). The results are shown in Fig 4A.

The duration of stimulation was calculated based on the return of EP density to 85% of normal values. ANOVA results showed an overall difference among the groups (F_3,56_ = 36.51, P<0.001). Post-hoc analysis showed that the perturbation duration was significantly longer with bilateral stimulation (p = 0.008, α = 0.0125) while there was no difference in the duration between left and right side stimulation (p = 0.29, α = 0.0125). The results are shown in Fig 4B.

### Vector analysis

**Fig 5:**
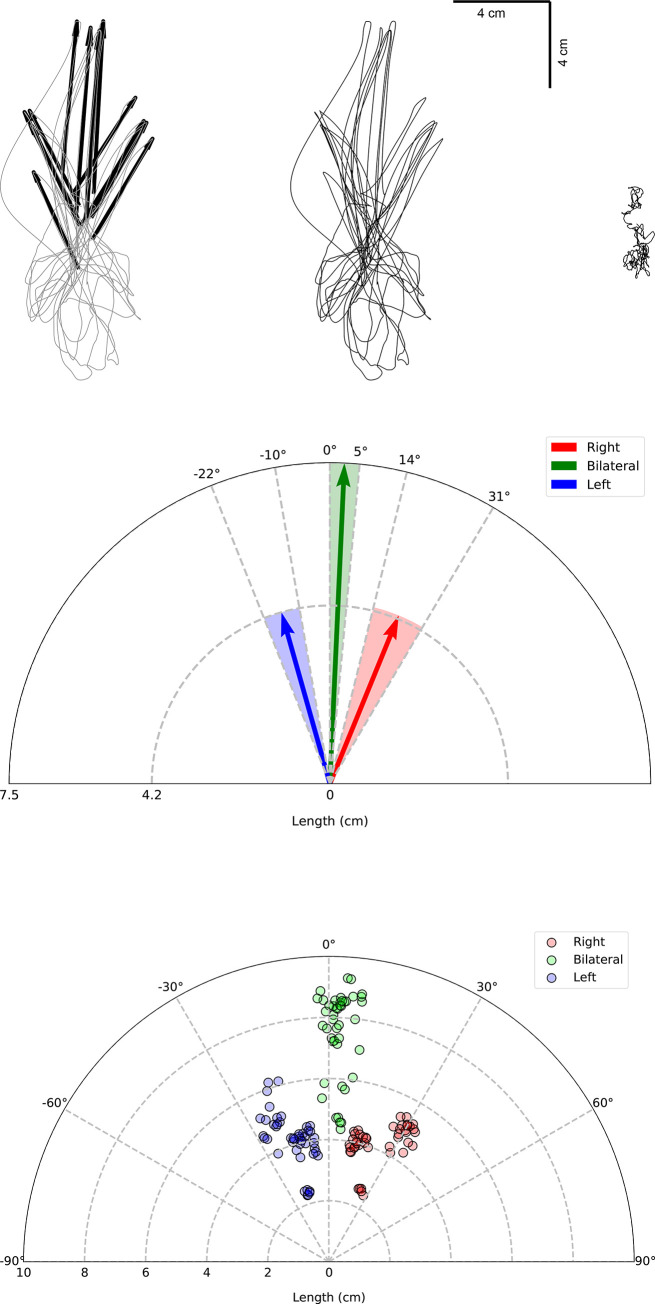
Vector analysis of perturbation. (A) Sample of a typical subject. From right to left: Thirty seconds of unperturbed COP, COP perturbation without vectors (For clarity), which took about 300 seconds, COP perturbation with vectors overlayed (same as the middle graph). (B) Vector analysis results for all subjects is shown in this graph. The shaded area represents the SD of each perturbation direction with vectors representing the direction and amplitude of perturbation. (C) Same as in figure B but instead of vectors, actual points are plotted. The five trials for each stimulation direction (left, right, both) were averaged for each subject to represent the response profile of that subject. These average points are plotted for all 15 subjects. There are, therefore, 45 points for each direction. A line connecting the center (0,0) to each point would constitute its vector.

The initial deflection in the postural sway was presented as a forward-direction vector. The size of the vector is an indicator of perturbation amplitude. The direction of the vector is an indicator of how the COP moved in response to perturbation. Fig 5A shows a sample of these vectors from a top view (looking from above over the forceplate area).

The three distinct directions of vectors, corresponding to the side of stimulation, are clearly observable. ANOVA results on the direction and length of the vectors showed an overall difference (F_3,56_ = 286.39, P<0.001 and F_3,56_ = 54.71, P<0.001, respectively). Post-hoc analysis showed significant change between any two comparisons in the directions (P<0.001). The length of the resultant vector for bilateral stimulation was significantly larger than the left and the right-side stimulation (P<0.001). There was no difference in the length of the vector between the two conditions of left and right-side stimulation (P = 0.39). The results are summarized in Fig 5B and 5C.

## Discussion

Simulation of loss of balance is a challenging laboratory experiment. Normal and skillful movements are relatively easy to replicate in labs, given that there is always a laboratory effect to change motor behavior [27]. However, examining an unskillful movement with an unpredictable nature is moving to a new level of difficulty in lab-based movement analysis. Therefore, in laboratory settings, a paradigm is defined to artificially trigger LOB response mechanisms and measure the reactions, or lack thereof.

The method presented here uses an artificial reflex protocol to cause muscle twitch and therefore, perturb balance. This is an experimental, lab-based approach to induce a controlled perturbation to standing posture and observe loss of balance and recovery mechanisms to bring COP back to its equilibrium. The brief twitch in postural muscles at the ankle joint (the hinge joint in the inverted pendulum model), is the cause of perturbation. Contraction of the same muscle group is also the first line of response to perturbation [28]. As such, inducing measurable amount of twitch and recording the EMG activity of muscle contraction is easily attainable in this method. This method is suitable for neuromechanical studies of posture and perturbation. This method, in all likelihood, triggers a less complicated control mechanism (rather than triggering multiple levels of responses).

Sway has been viewed as a search mechanism to find the limits of stability [25,29]. An interpretation of this hypothesis is that whenever the postural control system experiences instability or insecurity, it increases its search [30]. Our finding of an increase in EP density during perturbation, corroborates to this hypothesis and suggests that in a period of instability, the CNS switches faster from one instantaneous equilibrium point to another. The observation of the difference between left-side stimulation and bilateral stimulation needs more investigation, but we speculate that this could be related to side dominancy, as our subjects were all right leg dominant.

The density of EPS, therefore, might be regarded as a reasonable marker to examine the instability phase of a perturbation. Taken together, our perturbation-analysis technique provides a valuable method for inducing and quantifying perturbation in laboratory settings. Here we examined this method on a group of young, healthy individuals.

We yet, have to examine it on elderly people and/or people at high risk of falling to observe and report differences between fallers and non-fallers and also report the effect of aging on this observed control mechanism.

Therefore, our method not only provides biomechanical markers for quantifying perturbation, but also neural insights about motor programming at the level of final common pathway. This method does not claim any superiority over the already established methods or claims to solve inherent difficulties associated with the study of falling/loss of balance in a laboratory setting. Rather, it provides meaningful neuromechanical biomarkers with the potential for predicting risk of falling in different populations including neurological patients and elderly people. This method also has potential uses in clinical trial intervention studies. As a limitation to this study, we did not examine the method on other age ranges and therefore, cannot infer whether the density of EPs would be the same in other age ranges. We also did not compare this method to similar ones. We have not attempted to find any correlation between LOB induced by electrical stimulation and risk of falling assessment. This method is not designed to simulate or induce a fall. However, understanding the mechanisms or lack of certain reactions during this experimental procedure might be predictors of risk of fall. We aim to examine this method on a group of elderly people with no history of fall (non-fallers and a group of fallers to observe the differences in their reaction to this type of perturbation in terms of the density of EPs and the perturbation vector.

In summary, this paradigm provides a new method for investigating mechanism of loss of balance and has the ability to induce unilateral or bilateral perturbation. The method does not use any external perturbation source and has a faster time scale than mechanical perturbations. Future studies are needed to define the limits of normal values based on age.
